# Editorial: Explainable Artificial Intelligence (XAI) in Systems Neuroscience

**DOI:** 10.3389/fnsys.2021.766980

**Published:** 2021-10-29

**Authors:** Angela Lombardi, João Manuel R. S. Tavares, Sabina Tangaro

**Affiliations:** ^1^Dipartimento di Fisica, Università degli Studi di Bari Aldo Moro, Bari, Italy; ^2^Istituto Nazionale di Fisica Nucleare, Sezione di Bari, Bari, Italy; ^3^Departamento de Engenharia Mecânica, Faculdade de Engenharia, Instituto de Ciência e Inovação em Engenharia Mecânica e Engenharia Industrial, Universidade do Porto, Porto, Portugal; ^4^Dipartimento di Scienze del Suolo, della Pianta e degli Alimenti, Università degli Studi di Bari Aldo Moro, Bari, Italy

**Keywords:** XAI, explainable AI, neuroscience, deep learning, interpretability

In the last 10 years, we have experienced exceptional growth in the development of machine-learning-based (ML) algorithms for the analysis of different medical conditions and for developing clinical decision support systems. In particular, the availability of large datasets and the increasing complexity of both hardware and software systems have enabled the emergence of the new multidisciplinary field of computational neuroscience (Teeters et al., 2008). Sophisticated machine learning algorithms can be trained using brain imaging data to classify neurodegenerative disorders, detect neuropsichiatric conditions (Davatzikos, 2019), and perform accurate brain age prediction for the identification of novel functional and structural biomarkers for different diseases (Cole and Franke, 2017).

Deep learning (DL) models are increasingly used by scientific communities due to their higher accuracy and efficiency. Deep learning comprises different classes of algorithms that implements artificial neural networks with deep layers. These models have proved effectiveness in a wide range of applications since they can be used even with non-trivial relationships among the features of a predictive task and between the features and the outcomes. On the other hand, due to the high inner complexity of the algorithms, is often difficult to obtain insights into the workings of the deep learning models. Their “black-box” nature makes the models less trustworthy to physicians, thus hindering their expansion into real clinical settings (Ribeiro et al., 2016).

More recently, many efforts have been made to improve the interpretability of the decisions of machine learning algorithms. Specifically, the research area of Explainable Artificial Intelligence (XAI) has emerged, which aims to provide new methodologies and algorithms to enhance transparency and reliability to both the decisions made by predictive algorithms and the contributions and importance of individual features to the outcome (Guidotti et al., 2018; Gunning et al., 2019; Arrieta et al., 2020). Several works have demonstrated the central role of XAI methods for personalized medicine, including individualized interventions and targeted treatments (Fellous et al., 2019; Langlotz et al., 2019; Tjoa and Guan, 2020). However, such techniques have not yet been extensively explored in computational neuroscience.

In this Research Topic, we collected several original research works where different XAI techniques were embedded in both ML and DL algorithms for the extraction of reliable biomarkers from neuroimaging datasets for several predictive tasks.

Kim and Ye used 942 resting state fMRI scans from the preprocessed HCP dataset to train Graph Neural Networks (GNNs) for gender classification. They exploited information provided by graph architecture on functional connectivity networks by means of GNNs which comprise graph operations performed by deep neural networks and demonstrated that the gender classification method is able to effectively extrapolate state-of-the-art results by achieving high accuracy values. At the same time, the authors introduced an important mathematical formalization concerning the relationship between GNN and CNN. Based on this relationship, they used a saliency map visualization technique for CNN, i.e., the gradient-weighted class activation mapping (Grad-CAM) to visualize the important brain regions resulting from the classification task, overcoming the current limitation issue about the interpretability of the GNN architectures.

The gender classification task was also treated by Bučková et al.. The authors tested a deep convolutional neural network trained to identify biological gender from EEG recordings of a healthy cohort on another dataset of EEG data of 134 patients suffering from Major Depressive Disorder. In their work, they developed an explainable analysis to verify the discriminative power of beta-band power and test its effectiveness before and after the antidepressant treatment by highlithing the contribution of each electrode in order to clearly identify the final set of biomarkes.

The crucial role of XAI methods for clinical personalized analysis was explored in the work of Lopatina et al. where several XAI methods based on attribution maps (heatmaps) were used in conjunction with a CNN to both identify multiple sclerosis patients from 2D susceptibility-weighted imaging scans and highlight individual heatmaps indicating the contribution of a given voxel to the classification decision.

Lombardi et al. also showed how to use local XAI algorithms to extract personalized information about the importance of several brain morphological descriptors extracted from the MRI scans of a healthy cohort of subjects for the prediction of the biological age. The authors presented an explainable DL framework to evaluate the accuracy of the models while achieving high interpretability of the contribution of each brain morphological feature to the final predicted age They introduced two metrics (i.e., intra-consistency and inter-similarity) to compare different XAI methods and quantitatively establish their reliability in order to choose the most suitable for the age prediction task.

Varzandian et al. adopted the brain MRI scans of 1901 subjects to train a predictive model based on the apparent brain age and the chronological age to classify Alzheimer's disease patients. The authors developed a workflow to perform the regression and classification tasks maintaining the morphological semantics of the input space and providing a feature score to assess the specific contribution of each morphological region to the final outcome.

Finally, although the concept of interpretability has several implications, one of the most important regards the ability to understand also the errors and pitfalls of the ML and DL algorithms. Bae et al. focused their work on revealing misleading points that may arise from the pre-defined feature space. They used a DNN architecture to simulate four different problem scenarios such as the incorrect assessment of the feature selectivity, the use of features that act as confounding variables, the overestimation of the network feature representation and several misassumptions regarding the feature complexity.

In conclusion, all the works included in this Research Topic outline the potential effects of XAI techniques in different diagnostic scenarios and show how empirical studies could draw future directions for boosting XAI in real clinical applications.

## Author Contributions

All authors listed have made a substantial, direct and intellectual contribution to the work, and approved it for publication.

## Funding

This work was supported in part by the research project Biomarcatori di connettività cerebrale da imaging multimodale per la diagnosi precoce e stadiazione personalizzata di malattie neurodegenerative con metodi avanzati di intelligenza artificiale in ambiente di calcolo distribuito (project code 928A7C98) within the Program Research for Innovation -REFIN funded by Regione Puglia (Italy) in the framework of the POR Puglia FESR FSE 2014-2020 Asse X - Azione 10.4.

## Conflict of Interest

The authors declare that the research was conducted in the absence of any commercial or financial relationships that could be construed as a potential conflict of interest.

## Publisher's Note

All claims expressed in this article are solely those of the authors and do not necessarily represent those of their affiliated organizations, or those of the publisher, the editors and the reviewers. Any product that may be evaluated in this article, or claim that may be made by its manufacturer, is not guaranteed or endorsed by the publisher.
